# Surveillance and Molecular Characterization of SARS-CoV-2 Infection in Non-Human Hosts in Gujarat, India

**DOI:** 10.3390/ijerph192114391

**Published:** 2022-11-03

**Authors:** Dinesh Kumar, Sejalben P. Antiya, Sandipkumar S. Patel, Ramesh Pandit, Madhvi Joshi, Abhinava K. Mishra, Chaitanya G. Joshi, Arunkumar C. Patel

**Affiliations:** 1 Gujarat Biotechnology Research Centre (GBRC), Sector-11, Gandhinagar 382011, Gujarat, India; 2 Department of Veterinary Microbiology, College of Veterinary Science and Animal Husbandry, Sardarkrushinagar Campus, Kamdhenu University, Gandhinagar 382010, Gujarat, India; 3 Molecular, Cellular and Developmental Biology Department, University of California Santa Barbara, Santa Barbara, CA 93106, USA

**Keywords:** buffalo, canine, cattle, delta variant, RT-qPCR, SARS-CoV-2

## Abstract

Since December 2019, Severe Acute Respiratory Syndrome Coronavirus-2 (SARS-CoV-2) has been spreading worldwide, triggering one of the most challenging pandemics in the human population. In light of the reporting of this virus in domestic and wild animals from several parts of the world, a systematic surveillance study was conceptualized to detect SARS-CoV-2 among species of veterinary importance. Nasal and/or rectal samples of 413 animals (dogs n= 195, cattle n = 64, horses n = 42, goats n = 41, buffaloes n = 39, sheep n = 19, cats n = 6, camels n = 6, and a monkey n = 1) were collected from different places in the Gujarat state of India. RNA was extracted from the samples and subjected to RT-qPCR-based quantification of the target sequences in viral nucleoprotein (N), spike (S), and ORF1ab genes. A total of 95 (23.79%) animals were found positive, comprised of n = 67 (34.35%) dogs, n= 15 (23.43%) cattle, and n = 13 (33.33%) buffaloes. Whole SARS-CoV-2 genome sequencing was done from one sample (ID-A4N, from a dog), where 32 mutations, including 29 single-nucleotide variations (SNV) and 2 deletions, were detected. Among them, nine mutations were located in the receptor binding domain of the spike (S) protein. The consequent changes in the amino acid sequence revealed T19R, G142D, E156-, F157-, A222V, L452R, T478K, D614G, and P681R mutations in the S protein and D63G, R203M, and D377Y in the N protein. The lineage assigned to this SARS-CoV-2 sequence is B.1.617.2. Thus, the present study highlights the transmission of SARS-CoV-2 infection from human to animals and suggests being watchful for zoonosis.

## 1. Introduction

The member viruses of the family *Coronaviridae* are known to cause diseases in a wide variety of domestic and wild animals and humans because of their ability to jump the species barrier [1]. In the recent past, Severe Acute Respiratory Syndrome (SARS) and Middle East Respiratory Syndrome (MERS) diseases proved to be transmitted to humans through palm civets (*Paradoxurus hermaphroditus*) and camels (*Camelus dromedarius*), respectively [2]. In this connection, the recent pandemic of Coronavirus Infection Disease-2019 (COVID-19), caused by SARS-CoV-2, has spanned almost the whole human-inhabited world in a very short span of time and still continues. Though the disease has largely affected humans, it is also postulated to originate from animals, which has yet to be proven conclusively [3].

The causative virus belongs to the genus *betacoronavirus*, which also includes coronaviruses of horses, cattle, pigs, etc. Its single-stranded positive sense RNA genome is the largest among the RNA viruses and codes for four structural proteins, viz., the spike (S), envelope (E), membrane (M), and nucleocapsid (N) proteins, along with several nonstructural proteins. The viral spike (S) protein is the cell-binding ligand and determinant of the outcome of infection in the host [4,5]. The RNA nature of the genome enables the virus to undergo exceptionally high numbers of mutations, and its large size facilitates its permanent genomic accommodation for ‘better fit and survivability’. Compared to the initially detected virus strain in China in 2019; the currently circulating strains of SARS-CoV-2 have accumulated several additional mutations that affect the virulence, immune evasion capacity, human-to-human transmissibility, and possibly the host range [6]. Its taxonomic position and mutation ability accentuate the species barrier jumping ability of SARS-CoV-2 from animal to human and vice versa. 

In India, the first case of COVID-19 infection was detected in January 2020 [7], and thereafter, a highly devastating wave of the disease (denoted as the second wave) occurred during the period April–June 2021, which peaked with about four hundred thousand human cases per day (Ministry of Health and Family Welfare, Government of India website). Recently, in December 2021 to January 2022, the third wave was characterized by high infection and morbidity, but relatively little mortality has recently been seen. The second wave was attributed to the Delta (B.1.617.2) lineage, and the third was to the Omicron (B.1.1.529) lineage of the SARS-CoV-2. 

Meanwhile, few reports have been published to denote the presence of this virus in zoos and domestic animals, either as case reports or large-scale epidemiological studies from several parts of the world [8,9,10,11], including India [12]. In experimental animal models, varied susceptibility of this virus has been found in domestic and lab animals [13]. Therefore, it creates an additional possibility that the virus may use one or multiple animal species as its reservoir, and then, it will infect human from time to time in an endemic/epidemic manner. Even the reemergence of the disease in pandemic form is also possible, with a possible evolution of vaccine immunity evading the virus strains [14]. In addition to this, after the passing of more than two years of the disease, a large population of the country is now showing reluctance to maintain appropriate COVID-19 behaviors. All these circumstances compel the scientific community to undertake simultaneous and continuous surveillance of SARS-CoV-2 in animals, along with humans. 

Considering these facts, the present study has been conceptualized to detect SARS-CoV-2 among domestic animal species, viz., dogs, cattle, buffaloes, sheep, goats, camels, cats, and horses, in animal–human coinhabiting areas using RT-qPCR. In addition to this, a complete genome of SARS-CoV-2 was sequenced to determine the mutations and statuses of the associated variants. To the authors’ knowledge, this is the first systematic surveillance study of SARS-CoV-2 in animals in India, and the information generated will be of particular importance to trace the source of infection; which will ultimately help to contain the SARS-CoV-2 infection in animals and to develop effective control strategies. 

## 2. Materials and Methods

### 2.1. Location, Animals, and Sampling

**Figure 1:**
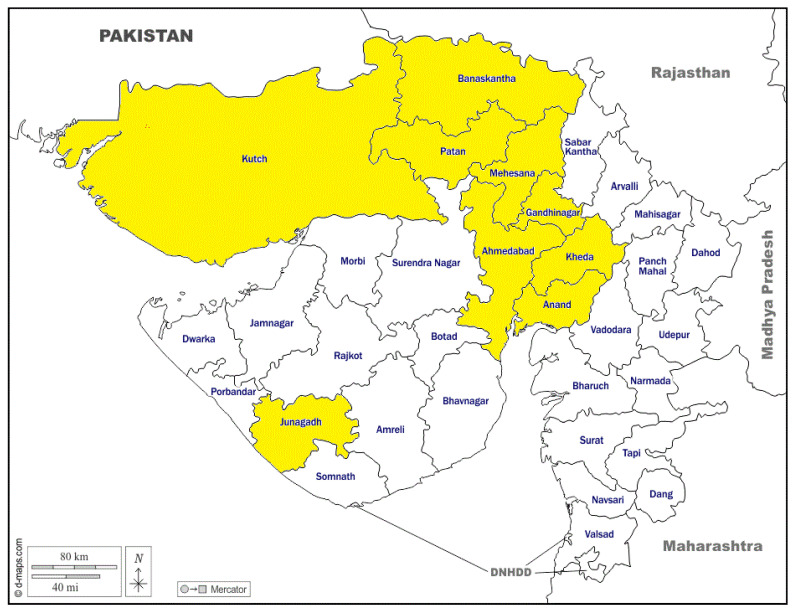
Map of Gujarat showing the locations of sample collections highlighted in yellow color.

The study was conducted from April 2021 to March 2022 in the north and central regions of the Gujarat state of India (Figure 1), which has an appreciable number of domestic and pet animals and is pioneering in a cooperative dairy setup. A total of 413 animals were screened for the presence of SARS-CoV-2 during the study period (Table 1), including dogs (n = 195), cattle (-n = 64), buffaloes (n = 39), goats (n = 41), sheep (n = 19), horses (n = 42), a monkey (n = 1), cats (n = 6), and camels (n = 6). Samples, viz., nasal (n = 412) and rectal swabs (n = 407), were collected in virus transport media (BeneSphera from Avantor Performance Materials India Ltd., Dehradoon, India) and transported to the laboratory under a cold chain. Dog sampling was done from a selected area where confirmed human COVID-19 cases have already been recorded. For the ruminant species, viz., cattle, buffaloes, sheep, and goats, five samples were randomly collected from different animal-rearing pockets coinciding with COVID-19 human infections. The horses and other samples were taken as per feasibility and availability in the study area. The details of the number of samples as per area and species involved are depicted in a tabulated form (Table 1). 

### 2.2. Virus RNA Isolation and RT-qPCR

The nasal and rectal swabs collected in VTM were processed for viral RNA extraction in Class II Biosafety cabinets with hard ducting using the QIAamp^®^ Viral RNA Mini Kit (Cat No. 52906, Qiagen, Germany) at the COVID-19 RT-PCR Lab (Indian Council of Medical Research accredited laboratory at the Department of Veterinary Microbiology, Sardarkrushinagar, Dantiwada, Gujarat, India) under strict biocontainment and biosafety measures. SARS-CoV-2 nucleic acid/RNA was detected using the CoviPath^TM^COVID-19 RT-PCR kit (Ref-A50780, Applied Biosystems, Banglore, India) in Applied Biosystem’s 7500 real-time PCR system. The nucleoprotein (N), spike (S), and ORF 1ab genes of SARS-CoV-2 were targeted to confirm the infection of SARS-CoV-2 in animals. The master mix and cyclic conditions were kept as per the manufacturer’s instructions, and the results were interpreted as positive if a minimum of two genes showed ≤35 Ct values. The RdRp gene, which is supposed to not be amplified in animal samples, ultimately helped us to rule out human contamination. 

### 2.3. Whole-Genome Sequencing

The representative samples that yielded low Ct values and, consequently, high viral RNA loads were further subjected to whole-genome sequencing using the IonGeneStudio S5 plus system and SARS-CoV-2 Research Panel (Thermo Fisher Scientific, Pleasanton, CA, USA). For this, the method we follow is the same as described in our previous study [15]. The data was analyzed using CLC genomics workbench v 12.0.3. The reads were mapped to the reference strain of SARS-CoV-2 (NC_045512.2) to determine the changes in the nucleotides and, consequently, amino acids. The amino acid profile was matched with pangolin V 3.1.7, and the WHO criteria for determination of the variant and lineage were assigned based on https://cov-lineages.org (accessed on 15 April 2022).

### 2.4. Phylogenetic Analysis

The SARS-CoV-2 genome recovered from the dog samples was analyzed for its phylogenetic placement using the Augur bioinformatic pipeline [16] with the reference genome SARS-CoV-2 NC_045512.2. Multiple-sequence alignment (MSA) was performed using MAFFT, while a maximum likelihood phylogenetic tree was reconstructed with the ultrafast bootstrap algorithm using IQ-TREE version 2 [17] on the local server with default parameters at the computational facility at GBRC, Gandhinagar, Gujarat. The phylogenetic tree was visualized in the Interactive Tree Of Life (iTOL) version 5 (https://itol.embl.de (accessed on 14 October 2022) webserver [18]. The additional complete SARS-CoV-2 genomes from human subjects and other animals (Supplementary Table S1) were obtained from the GISAID server [19].

## 3. Results

### 3.1. Sampling and Prevalence Data

A total of 413 animals were screened for the presence of SARS-CoV-2 during the study period (Table 1) from different places in the Gujarat state of India. Out of them, n = 95 (23.79%) animals were found to be positive during RT-qPCR. Among these 95 animals, respectively, 67/195 (34.35%), 15/64 (23.80%), and 13/39 (33.33%) dogs, cattle, and buffaloes were found to harbor the virus (Table 2). Both or either (nasal or rectal) sample from these animals were found positive during the RT-qPCR test. However, none of the samples from the goats (n = 41), sheep (n = 19), horses (n = 42), monkey (n = 1), cats (n = 6), or camels (n = 6) were found positive for SARS-CoV-2. 

### 3.2. Analysis of RT-qPCR Results

The difference in RT-qPCR positivity was also observed between the nasal and rectal swabs (Table 2), but the difference was statistically nonsignificant in the 2 × 2 chi-square test (*p* = 0.4178). Considering Ct value as a measure of the viral RNA load (Table 3), it was found that, among the positive nasal samples of the dogs, the Ct values ranged 27.63–34.98 ± 2.25 (mean 32.05), 28.40–34.96 ± 2.03 (mean 31.88), and 29.60–34.96 ± 1.39 (mean 32.9) for the N gene, ORF1ab gene, and S gene, respectively, whereas the positive rectal swabs of the dogs showed Ct values 27.89–34.97 ± 2.16 (mean 32.12), 28.97–34.93 ± 1.64 (mean 32.51), and 30.68–34.96 ± 1.19 (mean 33.37) for the N gene, ORF1ab gene, and S gene, respectively (n = 67). All positive nasal and rectal samples of the cattle (n = 15) and buffaloes (n = 13) showed Ct values above 30; however, the differences were not statistically significant. This could be a limitation due to the higher number of samples of dogs compared to cattle and buffaloes. 

### 3.3. Whole-Genome Sequencing and Variant Determination

A total of four dog nasal swab samples with low Ct values (<28) during RT-qPCR were subjected to whole SARS-CoV-2 genome sequencing using the Ion Torrent GeneStudio S5 system and SARS-CoV-2 research panel. However, only one sample (sample ID A4N) collected from Fayadi Canal, Johapura, Ahmedabad, yielded a good quality and interpretable sequencing data (Table 4). The genome sequencing data revealed that the virus had undergone 32 mutations from the original reference strain. Out of them, 29 were single-nucleotide substitutions and two were deletions. The nucleotide stretches AGTTCA and GA located at the 22,029 and 28,248 positions, respectively, were found to be deleted in the virus present in the sample. Further, nine out of the total of 32 mutations were located in the spike protein region. Out of them, one was a synonymous mutation (nt = 25,139) and one was a deletion (nt = 22,029). The consequent changes in the amino acid sequence revealed that the mutations occurred in the spike protein as T19R, G142D, E156-, F157-, A222V, L452R, T478K, D614G, and P681R. These are the designated mutations to classify the virus as the B.1.617.2 lineage delta variant, as defined by the WHO, whereas additional three mutations of the delta variant, viz., D63G, R203M, and D377Y, in the N genes were also present in the sequenced genome. Overall, based on pangolin V 3.1.7, the strain was categorized as B.1.617.2, and the scorpion call designated this as a delta variant (B.1.617.2-like). The functional consequences of these changes have been interpreted by the published literature (Table 5).

## 4. Discussion

Since the beginning of the pandemic, as on 12 April 2022, 12.24 hundred thousand confirmed human cases and 10,942 deaths were recorded from Gujarat (https://www.mygov.in/covid-19 (accessed on 12 April 2022 ); therefore, COVID-19 infection due to SARS-CoV-2 is, in principle, a human disease. However, the presence of this virus has been reported in several animal species [8], with [12,20] or without symptoms [11]. Previously, the virus has also been reported in pet animals [11] and captive animals [12] as case reports. Simultaneously, large-scale surveillance studies [9,10,21,22] have also been carried out and found the virus in animal populations, though at varied prevalence.

In the present work, though we screened the samples from nine species of animals, we were able to demonstrate the presence of SARS-CoV-2 RNA in dogs, cattle, and buffaloes only. The limitation of the present study was that there were less samples collected and processed for the majority of species, and it is always better to have samples from different geographical areas. To our knowledge, this is the first report about the demonstration of the virus in cattle and buffaloes, but this needs to be further confirmed with virus isolation and sequencing, though a large-scale surveillance was undertaken to detect COVID-19 infection in farm animals, viz., buffaloes, cattle, horses, pigs, sheep, goats, etc. in Italy [23], but none of the animals were found to be positive. Lawton et al. [24] reported 5.9% seropositivity in equines, though. Further, this was the first large-scale surveillance report about the presence of the SARS-CoV-2 virus in dogs from India. Here, we also recorded the approximate percentage prevalence recorded by Calvet et al. [22], but higher in comparison to previous studies of pet animals conducted in European countries [9], Spain [21], and Thailand [10]. This difference could partially be explained on the basis of the predominance of lineage B.1.617.2 (delta variant) at the time of the present study, which is reported to be more infectious than the prevalent alpha variant in Europe [9] and Thailand [10] at the time of their respective studies.

**Figure 2:**
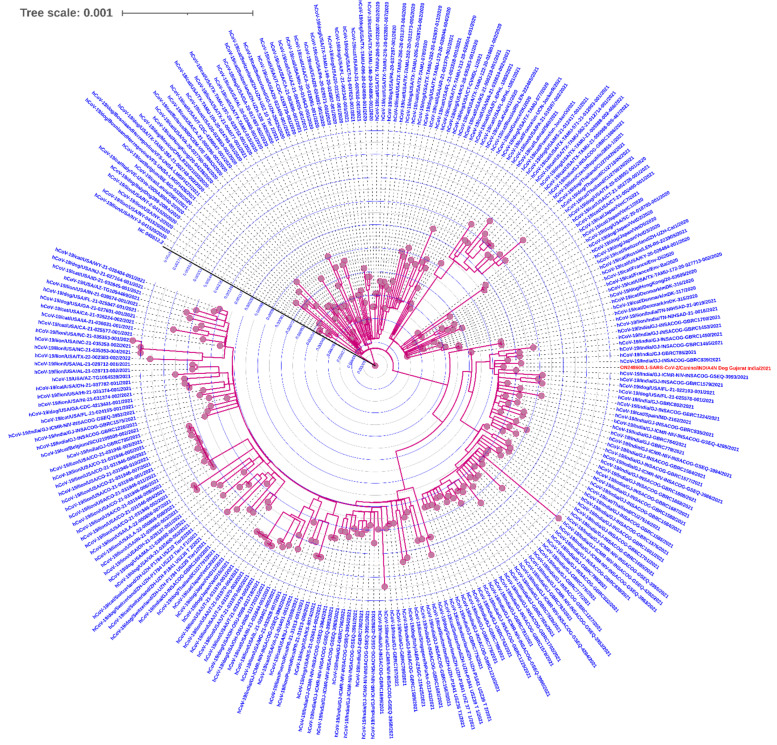
The phylogenetic placement of the A4N genome (orange) sequenced from a dog from Ahmedabad, Gujarat, India. The other genomes included were represented by *Canis lupus familiaris* (n= 50), human (n = 75), *Felis catus* (n = 81), and *Panthera leo* (n = 43), obtained from the GISAID webserver with the criteria of complete and high coverage, and additional metadata information is provided in Supplementary Table S1.

The maximum likelihood phylogenetic analysis (Figure 2) revealed the placement of the SARS-CoV-2 genome (A4N) from this study in the available genomes reported from domestic and wild animals reported by other researchers, as the metadata of the associated genomes is provided in Supplementary Table S1. Even during the early phase of the pandemic, pet animals such as dogs [9,22,25] were diagnosed and tested positive for SARS-CoV-2. Therefore, during the second wave, the probability of finding suspected animals positive was comparably higher, though the statistical analysis was limited due to factors beyond the scope of this research study. Yet, we provided formidable evidence for the importance of COVID-19 surveillance in domestic animals from Gujarat, India.

Though we also collected samples from horses, sheep, cats, camels, goats, and a monkey, none of these animals were found positive for SARS-CoV-2. Though members of the cat family such as domestic cats, ferrets [13], minks [26], and lions [12] have been reported to be important as reservoirs of this virus, develop respiratory symptoms, and are able to transmit it to other animals, positive samples from the cats could not be obtained due to rarity of cats as pet animals in the study area. Moreover, differences were observed with respect to the viral loads and number of positive rectal and nasal samples; this might be due to the use of a common kit for RNA isolation from both type of samples. Hence, it would be more appropriate to evaluate a specific kit for RNA isolation from fecal samples, which use high PCR inhibitors. 

This study indicates that the virus was secreted in nasal secretions and feces of dogs, cattle, and buffaloes. Further, all positive samples had Ct values ranging from approximately 28 to 39, which indicated a moderate load of the virus, which was not different from the approximate levels of the Ct values detected in animals by earlier workers [10,21]. These findings affirmed the fact stated by Shi et al. [13] that dogs are moderately susceptible animals for SARS-CoV-2. Still, even such a lesser viral load in a sample is enough to grow the virus on susceptible cell lines [21] and produce neutralizing antibodies. In contrast to humans, where only respiratory and oral droplets serve as the main source of infection, both respiratory and fecal secretions of animals may act as important sources of the infection and an additional source of the virus for environmental contamination and transmission [11]. Previously, our group also showed proof of surveillance of SARS-CoV-2 through wastewater systems [27] and, for the first time, reported the presence of SARS-CoV-2 delta variant B.1.617.2 in the wastewater system [15].

The presence of the delta variant in this study was confirmed by whole-genome sequencing. The whole-genome sequence data revealed that the mutations largely occurred in the ORF1a (n = 7), ORF1b (n = 4), and spike protein (n = 9) genes. Considering the spike protein is a major determinant of pathogenicity, out of nine mutations noted in the sequence, seven were nonsynonymous and produced consequent amino acid changes (Table 5). These substitutions and deletions in the spike protein of SARS-CoV-2 typically matched with the previously described changes in the SARS-CoV-2 delta variant [6,12,28]. Further, the functional significance of the changes was deciphered through earlier reports [6,12].

None of the RT-qPCR-positive animals in this study showed any symptoms related to COVID-19, and this finding was in concurrence with previous studies about dogs [9,11,21]. Possibly, only cats and members of the cat family show COVID-19-related symptoms, as Mishra et al. [12] showed the presence of respiratory symptoms due to the delta variant of COVID-19 in lions from India. 

In the present study, the majority of the positive animal samples were found to be collected during the second wave of the COVID-19 infection. Further, in the samples (n = 95) collected after the end of the second COVID-19 wave until March 2022, only two samples were found positive. One of the reasons for obtaining fewer positive samples after the second wave of COVID-19 might be the intensive human vaccinations in the study area. The vaccination drive for SARS-CoV-2 in India highlighted one of the largest global efforts in the fight against one of the deadliest pandemics in one of the most heterogenous and densely populated countries. These efforts helped to limit the active cases of COVID-19, which ultimately reduced the source of infection for animals. Hence, it can be concluded that the positive animals acquired the infection from humans, as well as human sources. The reverse transmission of the virus through the studied species was not observed/monitored in this study. This fact was substantiated by [9,10,21] through the study of animals under conditions of natural infection by humans and by Shi et al. [13] using an experimental infection model, though the role of cats and cat family members still requires further clarification under Indian conditions, as indicated by the perpetual infection of ferrets [13] and minks [26].

## 5. Conclusions

The present work described the surveillance of SARS-CoV-2 infection in non-human hosts in the Gujarat state of India through a systematic surveillance study. Cattle and buffaloes were found susceptible to infection of the virus, along with previously reported animals such as dogs, cats, and members of the cat family. The whole-genome sequencing of one sample also showed that the animal was infected with the delta B.1.1.617.2 variant and was also able to infect other animals. In summary, the present study highlighted the significance of SARS-CoV-2 surveillance in non-human hosts. This study was important; for instance, this type of transmission may pose a great threat to humans, as there is a high risk of the emergence of new SARS-CoV-2 variants.

## Figures and Tables

**Table 1:** Sampling details for COVID-19 surveillance in animals.

Name of District	Dog	Cattle	Buffalo	Goat	Sheep	Horse	Cat	Camel	Monkey	Total
Ahmadabad	114	-	-	-	-	-	-	-	-	114
Anand	17	-	-	-	-	1	-	-	-	18
Gandhinagar	39	26	13	-	-	-	-	-	-	78
Banaskantha	9	34	17	38	19	36	6	6	1	166
Patan	1	4	9	-	-	1	-	-	-	15
Kutch	15	-	-	-	-	1	-	-	-	16
Mehasana	-	-	-	3	-	1	-	-	-	4
Others *	-	-	-	-	-	2	-	-	-	2
Total	195	64	39	41	19	42	6	6	1	413

* Other places include the Sirohi District of Rajasthan and Saurashtra region of Gujarat.

**Table 2:** Results of the COVID-19 qPCR, according to species and sample.

Species	No of Animal Sampled	Nasal Swab Collected	Rectal Swab Collected	No. of Positive Samples in COVID-19 qPCR			
				Only Nasal Swab	Only Rectal Swab	Both Samples	Total
Dog	195	195	195	16	10	41	67
Cattle	64	64	63	5	2	8	15
Buffalo	39	39	39	4	3	6	13
Goat	41	40	37	-	-	-	-
Sheep	19	19	19	-	-	-	-
Horse	42	42	42	-	-	-	-
Monkey	1	1	1	-	-	-	-
Camel	6	6	5	-	-	-	-
Cat	6	6	6	-	-	-	-
Total	413	412	407	25	15	55	95

**Table 3:** Maximum and minimum Ct values obtained in the positive samples of different species during the qPCR of SARS-CoV-2.

Species (n = No. of Samples)	Type of Sample	N Gene (Cт)		ORF1ab (Cт)		S Gene (Cт)	
		Max–Min (± SD)	Mean	Max–Min (± SD)	Mean	Max–Min (± SD)	Mean
Dogs (n = 67)	Nasal	27.63–34.98 ± 2.25	32.05	28.40–34.96 ± 2.03	31.88	29.60–34.96 ± 1.39	32.9
	Rectal	27.89–34.97 ± 2.16	32.12	28.97–34.93 ± 1.64	32.51	30.68–34.96 ± 1.19	33.37
Cattle (n = 15)	Nasal	32.53–34.76 ± 0.72	34.13	32.26–34.94 ± 0.97	33.67	30.87–33.69 ± 0.92	32.40
	Rectal	33.78–35.00 ± 0.54	34.46	32.91–34.48 ± 0.57	33.8	30.30–34.95 ± 1.28	32.74
Buffaloes (n = 13)	Nasal	31.63–34.61 ± 1.05	33.16	31.74–34.84 ± 0.94	33.36	29.88–34.85 ± 1.59	32.78
	Rectal	29.00–34.93 ± 1.65	32.37	31.00–34.11 ± 2.16	32.92	29.79–34.35 ± 1.45	32.12

**Table 4:** Nucleotide changes observed in the whole-genome sequence of SARS-CoV-2 sequenced from a dog (Sample ID: A4N).

Reference Position	Reference	Allele	Count	Coverage	Frequency	Forward/Reverse Balance	Average Quality	Amino Acid Change
210	G	T	16,195	16,339	99.11	0.48	29.90	Synonymous
241	C	T	5204	5233	99.44	0.45	23.23	Synonymous
3037	C	T	11,467	12,330	93.00	0.38	28.40	Synonymous
5184	C	T	2593	2595	99.92	0.48	29.64	Pro1640Leu
5584	A	G	712	1809	39.35	0.50	31.35	Synonymous
9891	C	T	6039	6047	99.86	0.49	30.39	Ala3209Val
11,418	T	C	7924	7980	99.29	0.48	29.17	Val3718Ala
11,514	C	T	4237	4281	98.97	0.45	30.47	Thr3750Ile
13,019	C	T	2598	2607	99.65	0.47	31.25	Synonymous
14,408	C	T	2845	3822	74.43	0.49	30.01	Pro4715Leu
15,451	G	A	16,545	16,645	99.39	0.47	31.40	Gly5063Ser
15,919	G	T	6848	6868	99.70	0.45	32.26	Val5219Leu
16,466	C	T	10,720	10,735	99.86	0.21	31.83	Pro5401Leu
21,618	C	G	6431	6438	99.89	0.49	30.68	Thr19Arg
21,987	G	A	3751	3946	95.05	0.47	30.27	Gly142Asp
22,029	AGTTCA	-	3379	3685	91.70	0.50	28.26	Synonymous
22,227	C	T	1562	1591	98.18	0.49	25.47	Ala222Val
22,917	T	G	7672	7719	99.39	0.47	30.04	Leu452Arg
22,995	C	A	2725	2779	98.06	0.45	32.79	Thr478Lys
23,403	A	G	10,686	10,759	99.32	0.49	27.51	Asp614Gly
23,604	C	G	19,277	19,301	99.87	0.47	32.46	Pro681Arg
25,139	T	C	9114	9125	99.88	0.43	32.48	Synonymous
25,469	C	T	8791	8844	99.40	0.48	27.93	Ser26Leu
26,767	T	C	5947	5949	99.97	0.47	29.88	Ile82Thr
27,638	T	C	8633	8692	99.32	0.47	30.96	Val82Ala
27,752	C	T	6990	6993	99.96	0.45	30.07	Thr120Ile
28,248	GA	-	7439	7614	97.70	0.48	21.41	Synonymous
28,271	A	-	14,715	14,950	98.43	0.46	24.47	Synonymous
28,461	A	G	8647	8651	99.95	0.46	29.38	Asp63Gly
28,881	G	T	4107	4127	99.51	0.45	30.01	Arg203Met
29,402	G	T	5132	5192	98.84	0.49	27.77	Asp377Tyr
29,742	G	T	889	889	100.0	0.45	31.86	Synonymous

**Table 5:** Effects of the mutations observed in the sequence of the spike (S) protein of one SARS-CoV-2 genome from a dog (sample ID: A4N) in comparison to the reference strain.

Nucleotide Position	Nucleotide in Test Strain	Nucleotide in Reference Strain	Type of Mutation	Amino Acid Change	Possible Outcome of Mutation
21,618	C	G	SNV	Thr19Arg	Removes a potential N-glycosylation site that might also affect antigenic and other properties of this strain
21,987	G	A	SNV	Gly142Asp	
22,029	AGTTCA	-	Deletion		Possible deletion of antibody recognition site at amino acid position 156–157
22,227	C	T	SNV	Ala222Val	-
22,917	T	G	SNV	Leu452Arg	Host and other changes; antigenic drift; antibody recognition sites
22,995	C	A	SNV	Thr478Lys	Host and other changes; antigenic drift; host surface receptor binding; antibody recognition sites; viral oligomerization interfaces
23,403	A	G	SNV	Asp614Gly	Antigenic drift; virulence and host change; ligand binding; viral oligomerization interfaces
23,604	C	G	SNV	Pro681Arg	Increased rate of membrane fusion, internalization, and thus better transmissibility
25,139	T	C	SNV	-	-

## Data Availability

The whole-genome sequence of SARS-CoV-2 obtained for this research study was submitted to NCBI with accession no. ON248600.1 (https://www.ncbi.nlm.nih.gov/nuccore/ON248600.1/ (accessed on 18 April 2022).

## Supplementary Materials

The following supporting information can be downloaded at: https://www.mdpi.com/article/10.3390/ijerph192114391/s1; Table S1: Details of the SARS-CoV-2 genomes analysed in the phylogenetic tree.
